# Pterostilbene Induces Apoptosis in Awakening Quiescent Prostate Cancer Cells by Upregulating C/EBP-β-Mediated SOD2 Transcription

**DOI:** 10.7150/ijbs.106219

**Published:** 2025-05-07

**Authors:** Zhichao Xi, Mengfan Liu, Xue Jiang, Jiling Feng, Rongchen Dai, Wan Najbah Nik Nabil, Xueyang Sun, Jiayi Chen, Hangui Ren, Juan Zhang, Qihan Dong, Man Yuan, Yang Li, Hongxi Xu

**Affiliations:** 1 School of Pharmacy, Shanghai University of Traditional Chinese Medicine, No. 1200, Cailun Road, Shanghai 201203, China; 2 Engineering Research Center of Shanghai Colleges for TCM New Drug Discovery, No. 1200, Cailun Road, Shanghai 201203, China; 3 Precision Research Center for Refractory Diseases, Institute for Clinical Research, Shanghai General Hospital, Shanghai Jiao Tong University School of Medicine, Shanghai 201620, China; 4 National Pharmaceutical Regulatory Agency, Ministry of Health, Lot 36, Jalan Universiti, Petaling Jaya, Selangor 46200, Malaysia; 5 School of Chinese Medicine, Faculty of Medicine, The Chinese University of Hong Kong, Shatin, N.T., Hong Kong SAR 999077, China; 6 Chinese Medicine Anti-Cancer Evaluation Program, Greg Brown Laboratory, Central Clinical School and Charles Perkins Centre, Faculty of Medicine and Health, The University of Sydney, Sydney, NSW 2006, Australia; 7 Department of Endocrinology, Royal Prince Alfred Hospital, Sydney, NSW 2050, Australia

**Keywords:** Quiescent cancer cells, Prostate cancer recurrence, SOD2, C/EBP-β, Pterostilbene, Apoptosis

## Abstract

Quiescent cancer cells (QCCs) are known to resist chemoradiotherapy, evade immune surveillance and have the potential to drive recurrence years after initial treatment. However, the key regulators of QCC survival during reactivation remain unclear. This study revealed that superoxide dismutase 2 (SOD2) levels are significantly greater in quiescent prostate cancer (PCa) cells than in proliferative cells. SOD2 overexpression induces apoptosis in awakening quiescent PCa cells, whereas its knockdown promotes reactivation. Elevated SOD2 also suppresses recurrent tumor growth by quiescent PCa cells and prolongs survival. Pterostilbene (PTE), a natural compound, preferentially induces apoptosis in quiescent PCa cells during awakening and reduces their long-term proliferative capacity by upregulating SOD2. Additionally, PTE inhibits tumorigenesis and significantly reduces the growth of quiescent PCa cells without apparent toxicity. Further mechanistic studies revealed that CCAAT/enhancer-binding protein beta (C/EBP-β) is critical for PTE-mediated SOD2 upregulation by enhancing SOD2 transcription. C/EBP-β knockdown significantly reduces PTE-induced apoptosis in awakening quiescent PCa cells. Clinical analysis revealed a positive correlation between *CEBPB* and *SOD2*, with low C/EBP-β expression linked to poor prognosis. Overall, the C/EBP-β-SOD2 pathway is crucial for eliminating awakening quiescent PCa cells and highlights PTE as a promising agent for preventing PCa recurrence.

## Introduction

Despite considerable advances in primary cancer therapies, a significant proportion of patients still experience late recurrences following prolonged, disease-free intervals 1. Quiescent cancer cells (QCCs) experience a reversible arrest in the G_0_ phase of the cell cycle and pose a significant therapeutic challenge, as they are clinically undetectable, resistant to chemotherapy and immune privileged, allowing them to survive for years or even decades 2, 3. When QCCs exit the G_0_ phase, their cell cycle reentry (also termed “awakening”) restores proliferative capacity, thereby driving tumor recurrence 4. Several signaling pathways have been implicated in the transition between quiescence and proliferation in cancer cells. For example, the FBXW7-c-Myc axis and fibroblasts-derived collagen-I promote cancer cells exit from quiescence 5-7, whereas factors such as DYRK1B, Axl tyrosine kinase, and integrins inhibit QCC reactivation and enhance chemotherapy sensitivity 8-10. Moreover, STING suppresses QCC-driven metastatic recurrence by establishing an immunosuppressive microenvironment 11. Despite these investigations, the key regulators that sustain QCC survival during reawakening remain elusive.

Our previous proteomic and transcriptomic analyses identified superoxide dismutase 2 (SOD2) as the most upregulated molecule in quiescent prostate cancer (PCa) cells. Clinical data further reveal that reduced SOD2 expression correlates with poorer survival and higher recurrence rates in PCa patients, emphasizing its significance as a key regulator of QCC reactivation. In quiescent cells, elevated SOD2 facilitate the transition of mouse embryonic fibroblasts into quiescence, a process associated with SOD2-mediated alterations in glucose metabolism 12-14. Moreover, CCAAT/enhancer-binding protein beta (C/EBP-β), one of the primary transcription factors regulating SOD2 expression 15-17, is essential for maintaining the noncycling state of satellite cells and fibroblasts, with its loss promoting their exit from quiescence 18, 19. Although current studies implicate both C/EBP-β and SOD2 in the transition between quiescence and proliferation, the precise mechanism in the reactivation process of QCCs remains to be fully elucidated.

In this study, we demonstrated the detrimental role of SOD2 in awakening process. Notably, forced elevation of SOD2 induces apoptosis in awakening QCCs, while its knockdown accelerates cell cycle reentry. Pterostilbene (PTE) is a potent SOD2 activator that is commonly found in blueberries, grapes and herbal medicines 20, 21. It significantly induces apoptosis in awakening PCa cells and suppresses recurrent tumor growth without apparent toxicity. Further mechanistic studies revealed that C/EBP-β plays a pivotal role in PTE-mediated SOD2 upregulation by increasing its transcription. The present study provides new insights into the role of the C/EBP-β-SOD2 axis in eradicating awakening quiescent PCa cells and highlights PTE as a promising agent for preventing PCa recurrence.

## Results

### PTE eradicates awakening quiescent PCa cells by inducing apoptosis

**Figure 1:**
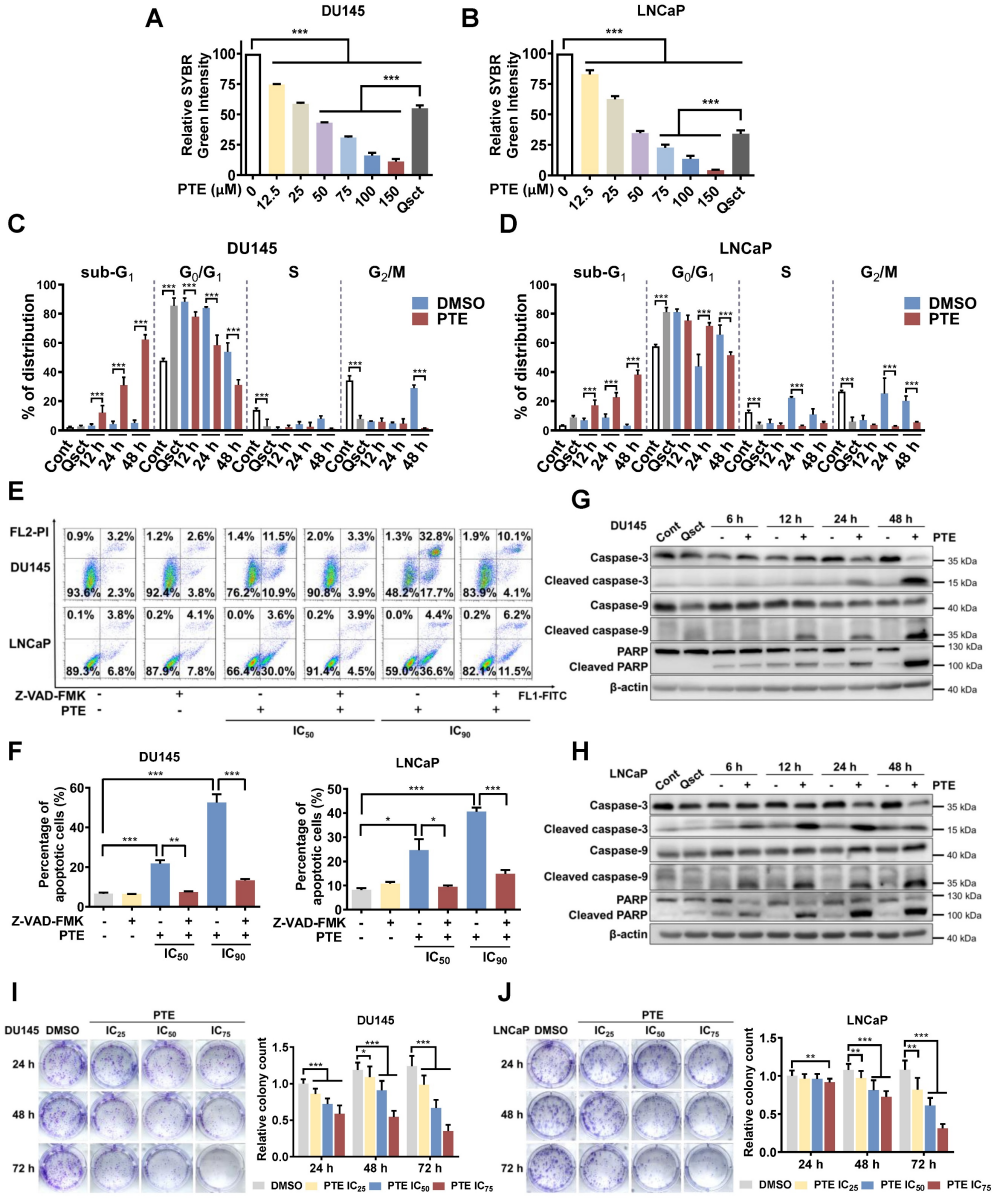
** PTE eradicates awakening quiescent PCa cells by inducing apoptosis. A, B** Quiescent DU145 (**A**) and LNCaP (**B**) cells were induced to re-enter the cell cycle, then exposed to various concentrations of PTE for 72 h. DNA content was subsequently measured using a SYBR Green assay.** C, D** Flow cytometric analysis with PI staining was performed to evaluate the cell cycle phase distributions in DU145 (**C**) and LNCaP (**D**) cells in the proliferative, quiescent, and cell cycle reentry phases, with or without PTE (IC_90_), at the indicated time points. **E, F** Quiescent DU145 and LNCaP cells were induced to re-enter the cell cycle with DMSO, PTE (IC_50_ and IC_90_) or 50 μM Z-VAD-FMK, either alone or in combination, for 48 h. Representative images (**E**) and quantification data of the percentage of apoptotic cells (**F**) were determined by flow cytometry with Annexin V-FITC/PI staining. **G, H** Western blotting was used to assess the protein expression levels of (cleaved-)caspase-3, (cleaved-)caspase-9, and (cleaved-)PARP in DU145 **(G)** and LNCaP **(H)** cells after PTE (IC_90_) treatment at the indicated times, with β-actin serving as the loading control. **I, J** Quiescent DU145 and LNCaP cells were induced to resume proliferation with or without PTE (IC_25_, IC_50_ and IC_75_) for 24, 48 and 72 h. Afterward, cells were cultured in PTE-free complete medium for 14 days and then stained with crystal violet. Representative images and colony quantification data of DU145 **(I)** and LNCaP **(J)** cells are presented. 'Cont' refers to proliferative control cells, and 'Qsct' indicates quiescent cells. The data are presented as the means ± standard deviations from three biological replicates. One-way ANOVA was applied to panels A and B; two-way ANOVA to panels C, D, I, and J; and a *t*‑test to panel F. ^*^P < 0.05, ^**^P < 0.01, ^***^P < 0.001 *versus* the indicated groups.

Experimental quiescence was induced in DU145 and LNCaP cells by withdrawing serum for 7 days. Subsequently, reintroducing serum triggered cell cycle re-entry and the reactivation of QCCs 5, 22. DNA resynthesis is a hallmark of quiescent cancer cells resuming proliferation 23. To assess changes in DNA content during the awakening of quiescent PCa cells with or without PTE, we performed a SYBR Green assay. Seventy-two hours after serum replenishment, PTE significantly reduced the DNA content in awakening PCa cells in a dose-dependent manner compared with the DMSO control group (Fig. 1A, B). Notably, following treatment with 75 μM PTE, the DNA content was even lower than that at baseline, suggesting that PTE induced death in quiescent PCa cells during reawakening. The inhibitory concentration (IC) values of PTE (IC_25_, IC_50_, IC_75_ and IC_90_) in awakening quiescent PCa cells were calculated (Table 1).

We further examined the cell cycle distribution during reactivation by employing PI-stained cells and flow cytometry. After 7 days of serum withdrawal, 85% of the DU145 cells and 81% of the LNCaP cells were in the G_0_/G_1_ phase, whereas 47% and 57% were in complete medium, respectively (Fig. 1C, D). Co-staining with Hoechst 33258 and Pyronin Y confirmed a pronounced increase in G_0_-phase cells in both PCa cell lines during quiescence (Fig. S1A, B). Twenty-four hours after release from the quiescent stage, PCa cells in the control group successfully re-entered the S and G2/M phases without significant accumulation in sub-G_1_. However, PTE significantly elevated the sub-G_1_ proportion within 48 h of reactivation compared with DMSO treatment (Fig. 1C, D; Fig. S1C). Annexin V-FITC/PI staining further revealed that PTE induced apoptosis in a dose-dependent manner, an effect that was substantially reversed by the pancaspase inhibitor Z-VAD-FMK (Fig. 1E, F). PTE activated caspase-3, caspase-9 and PARP in awakening quiescent PCa cells (Fig. 1G, H). Additionally, PTE treatment significantly diminished the mitochondrial membrane potential in these cells, corroborating the impairment of mitochondrial function (Fig. S1D). Notably, the same PTE treatment for 48 h resulted in less than 10% apoptosis in proliferative PCa cells (Fig. S1E).

To evaluate PTE's long-term inhibitory effect on awakening PCa cells, quiescent DU145 and LNCaP cells were treated with DMSO or PTE at the IC_25_, IC_50_ and IC_75_ concentrations for 24, 48 and 72 h during reactivation, followed by an additional 14 days of culture without PTE. Compared with the control, PTE markedly decreased colony formation in a dose- and time-dependent manner (Fig. 1I, J). Overall, these findings suggest that PTE preferentially induces apoptosis in PCa cells during their awakening from quiescence and reduces their long-term reproliferative ability.

### SOD2 overexpression induces apoptosis in awakening quiescent PCa cells

**Figure 2:**
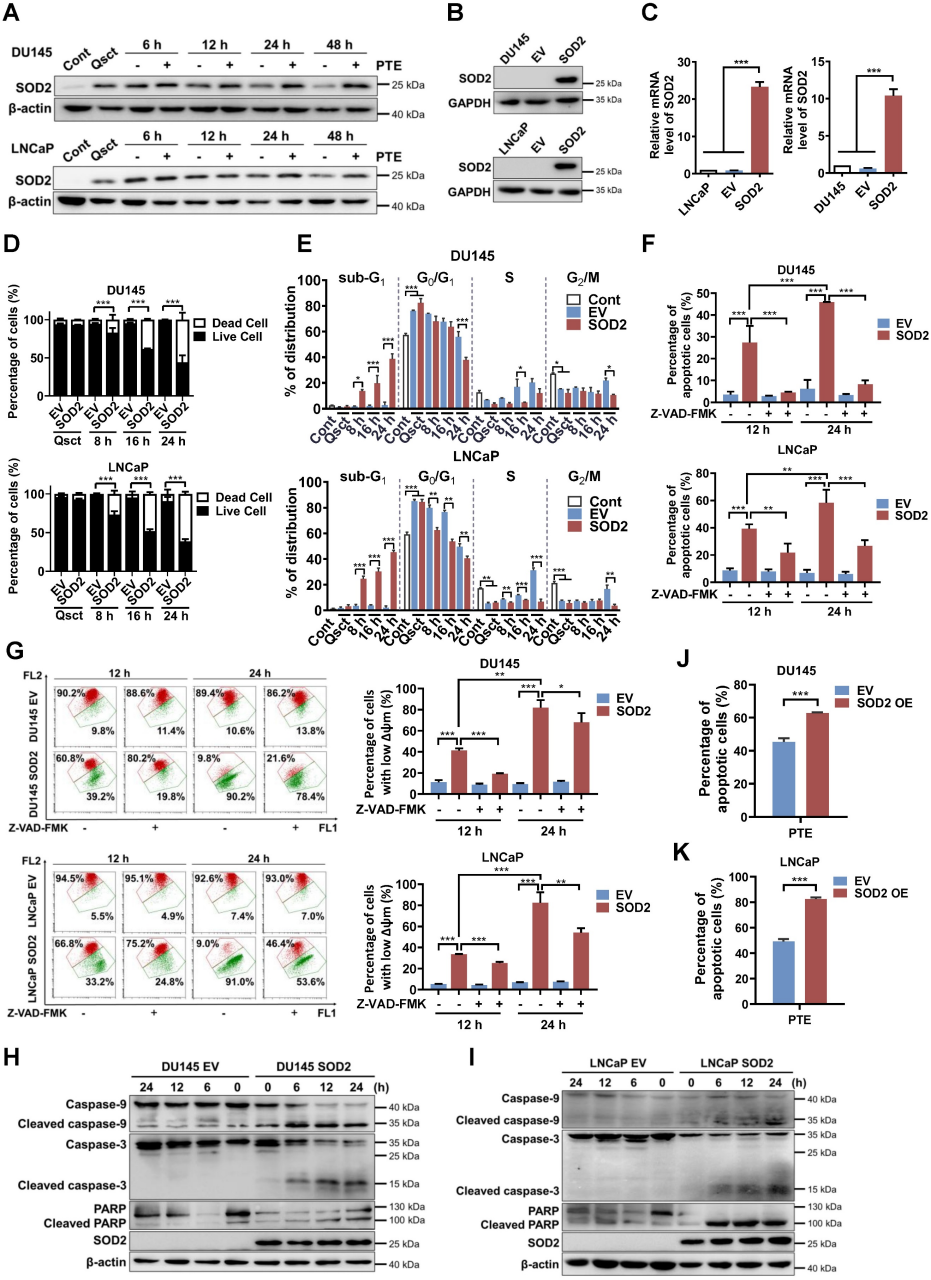
** SOD2 overexpression induces apoptosis in awakening quiescent PCa cells. A** The levels of SOD2 protein in PTE (IC_90_)-treated DU145 and LNCaP cells during cell cycle reentry at the indicated time points. **B, C** Protein **(B)** and mRNA **(C)** expression levels of SOD2 in DU145 and LNCaP cells stably transfected with either a SOD2-overexpression plasmid (SOD2) or a parental empty vector (EV). GAPDH and TBP were used as loading controls for western blotting and RT‒PCR, respectively. **D, E** Quiescent EV and SOD2-overexpressing DU145 and LNCaP cells were induced to re-enter the cell cycle. Cell viability **(D)** and cell cycle distribution **(E)** were assessed by flow cytometry using PI staining at the indicated intervals. **F, G** Following release from quiescence, EV and SOD2-overexpressing PCa cells were treated with or without Z-VAD-FMK (50 μM) for 12 or 24 h. Apoptotic cells **(F)** and the mitochondrial membrane potential **(G)** were measured by flow cytometry with Annexin V-FITC/PI and JC-1 staining, respectively.** H, I** Expression of apoptosis-related proteins in quiescent EV and SOD2-overexpressing DU145 **(H)** and LNCaP **(I)** cells during cell cycle reentry, with β-actin used as the loading control. **J, K** Apoptosis in SOD2-overexpressing DU145 **(J)** and LNCaP **(K)** cells, along with their EV control cells, was assessed by flow cytometry with Annexin V-FITC/PI staining after 48 h of PTE (IC_90_) treatment during cell cycle reentry. 'Cont' refers to proliferative control cells, and 'Qsct' indicates quiescent cells. The data are presented as means ± standard deviations from three individual experiments. ^*^P < 0.05, ^**^P < 0.01, ^***^P < 0.001 compared with the indicated groups.

Our previous multi-omics analyses identified SOD2 as the most upregulated molecule in quiescent PCa cells, we verified its expression during the reactivation process. In quiescent PCa cells, SOD2 protein and mRNA levels were significantly elevated than those in proliferative cancer cells, and these levels decreased during the awakening process (Fig. S2A-D). Interestingly, PTE treatment hindered the decrease in SOD2 protein levels in awakening PCa cells during cell cycle reentry (Fig. 2A). These observations indicate that SOD2 may have a regulatory function in the reactivation of quiescent PCa cells. Additionally, increased levels of phospho-Rb (Ser807/811) and decreased levels of p27 indicate progression of cell cycle reactivation, confirming the successful establishment of the quiescent model 5, 24 (Fig. S2A, B).

The primary function of SOD2 is to scavenge superoxide anion radicals and reduce mitochondrial ROS, thereby protecting against oxidative-induced cell death 25, 26. We first investigated the changes in mitochondrial ROS levels in response to PTE-induced SOD2 upregulation and apoptosis. Interestingly, we observed that the elevated SOD2 levels in quiescent PCa cells did not result in lower mitochondrial ROS levels than those in proliferative cells (Fig. S3A, B). PTE treatment increased mitochondrial ROS levels in quiescent PCa cells as they re-entered the cell cycle (Fig. S3C, D). Notably, the mitochondrial ROS scavenger, mitoTEMPO, neither reduced ROS levels nor prevented PTE-induced apoptosis. These findings suggest that PTE-induced SOD2 promotes apoptosis independently of mitochondrial ROS accumulation (Fig. S3C-F).

We then investigated whether SOD2 overexpression could inhibit the awakening of quiescent PCa cells. DU145 and LNCaP cells were stably transfected with a full-length SOD2 cDNA construct or an empty vector (EV). This transfection significantly elevated SOD2 protein and mRNA levels (Fig. 2B, C). Notably, 90.7% of EV PCa cells remained viable 24 h after reentry into the cell cycle, whereas SOD2 overexpression induced approximately 58.5% and 61.4% cell death in reactivated DU145 cells and LNCaP cells, respectively (Fig. 2D). Consistently, SOD2 overexpression increased the sub-G_1_ population as early as 8 h after reactivation in awakening PCa cells compared with EV PCa cells (Fig. 2E).

Further analysis using Annexin V-FITC/PI staining revealed that SOD2 overexpression significantly induced apoptosis during the reactivation of quiescent DU145 and LNCaP cells, and this effect was significantly reversed by Z-VAD-FMK treatment (Fig. 2F, Fig. S4A, B). Additionally, SOD2-overexpressing PCa cells presented a loss of mitochondrial membrane potential following cell cycle reentry, which was mitigated by Z-VAD-FMK, indicating the occurrence of mitochondria-dependent apoptosis (Fig. 2G). Caspase-3, caspase-9 and PARP were activated in SOD2-overexpressing PCa cells upon exiting quiescence (Fig. 2H, I). Importantly, the combination of SOD2 overexpression and PTE treatment further increased apoptosis in awakening quiescent PCa cells (Fig. 2J, K; Fig. S4C). These findings suggest that SOD2 is integral to the reactivation process and could be a promising therapeutic target for preventing PCa recurrence.

### SOD2 overexpression suppresses the growth of recurrent tumors from quiescent DU145 cells and prolongs mouse survival

**Figure 3:**
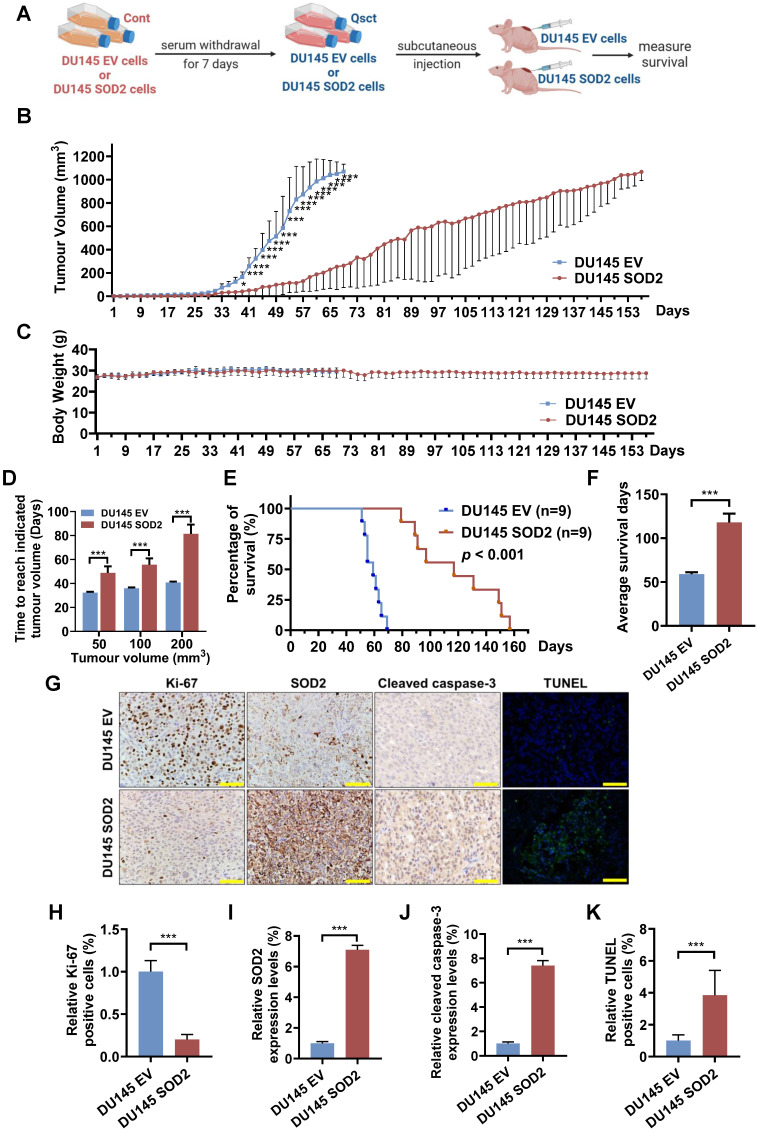
** SOD2 overexpression suppresses the tumorigenicity of quiescent DU145 cells and prolongs the mouse survival. A** Schematic diagram of the mouse model for recurrent PCa using quiescent DU145 EV or DU145 SOD2 cells. **B, C** Tumor volumes (**B**) and mouse body weights (**C**) were measured every other day, with euthanasia performed when tumor size reached 1000 mm^3^. **D** The average times for tumors in the DU145 EV and DU145 SOD2 groups to reach volumes of 50, 100, and 200 mm^3^.** E, F** Survival curve analysis (**E**) and quantification data (**F**) showing the overall survival probability of the mice in each group. **G-K** For immunohistochemical analysis, three randomly selected views from each section (n = 3 per group) were quantitatively analyzed using ImageJ software. A representative image is shown in panel **G**, and quantification data for Ki‑67, SOD2, cleaved caspase‑3, and TUNEL staining in resected tumors are provided in panels **H-K**. Scale bar: 50 μm. The data are presented as the means ± standard deviations. ^*^P < 0.05, ^***^P < 0.001 *versus* the control group or indicated group.

To evaluate the effect of SOD2 overexpression on the growth of recurrent tumors from quiescent PCa cells *in vivo*, quiescent control (DU145 EV) cells and SOD2-overexpressing (DU145 SOD2) cells were inoculated subcutaneously into nude mice (Fig. 3A). The growth rate of recurrent tumors in the SOD2-overexpressing group was significantly slower than that in the EV control group (Fig. 3B). No notable differences in body weight were observed between the two groups (Fig. 3C). The average times required to reach tumor volumes of 50, 100 and 200 mm³ were substantially prolonged in the SOD2-overexpressing group (Fig. 3D). Survival analysis revealed that SOD2 overexpression nearly doubled survival days relative to EV control group (Fig. 3E, F). Additionally, tumors from SOD2 overexpression group maintained high SOD2 levels, exhibited fewer Ki-67-positive cells and higher amount of cleaved caspase-3- and TUNEL-positive cells (Fig. 3G-K). These *in vivo* results demonstrate that elevated SOD2 expression suppresses the growth of recurrent tumors from quiescent PCa cells and prolongs survival.

### Knockdown of SOD2 promotes the awakening of quiescent PCa cells and reduces the apoptotic effects of PTE

**Figure 4:**
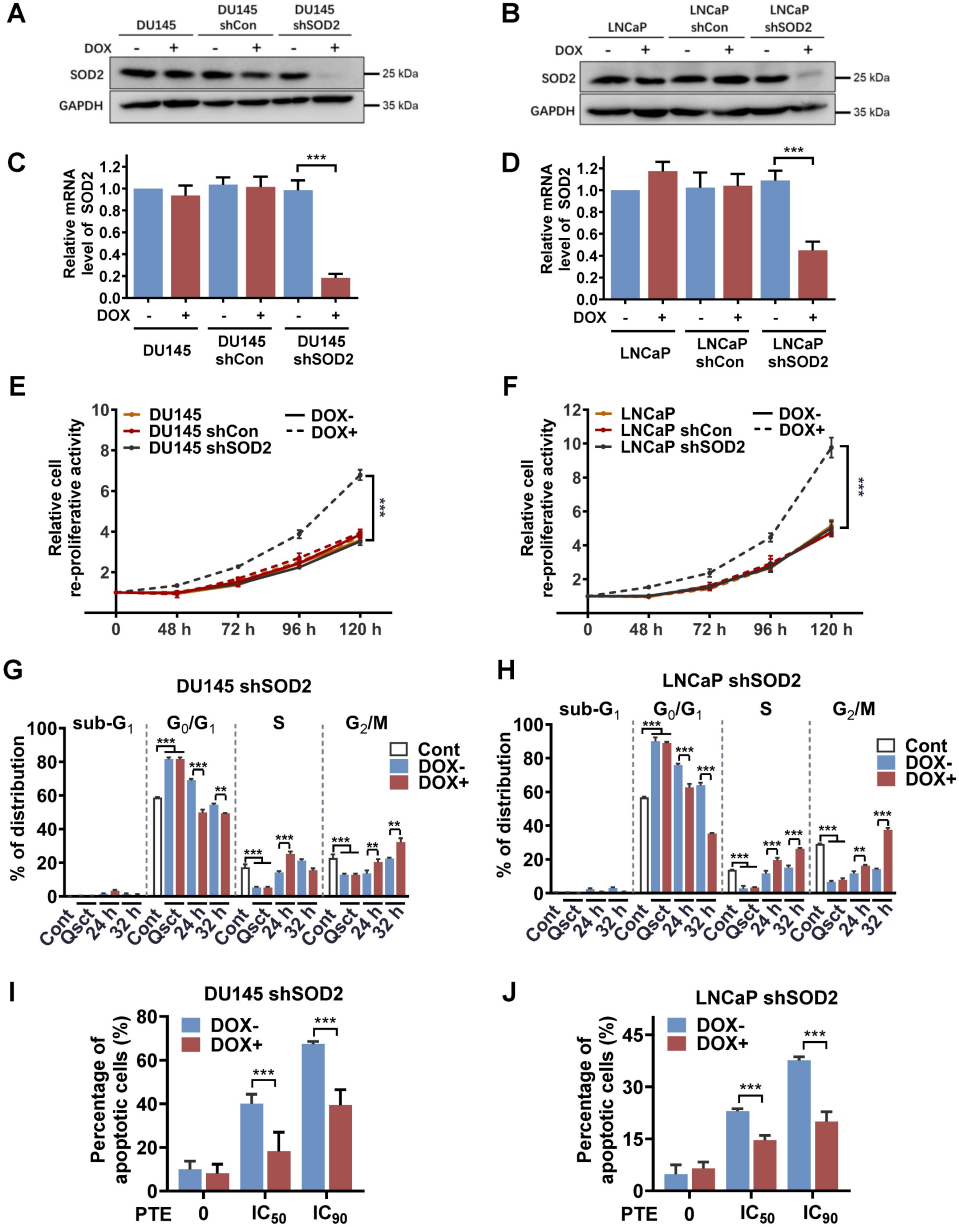
**Knockdown of SOD2 promotes cell cycle reentry in quiescent PCa cells. A-D** SOD2 protein and mRNA levels in nontransfected, DOX-inducible shCon or shSOD2 plasmid-transfected DU145 **(A, C)** and LNCaP **(B, D)** cells were analyzed by western blotting and RT‒PCR. **E, F** The reproliferative activity of the indicated cells during reactivation was assessed using a Trypan blue viability assay for 0‒120 h (normalized to 0 h). The cells were treated with DOX (4 μg/ml) for 48 h prior to reactivation. **G, H** Quiescent DU145 shSOD2 **(G)** and LNCaP shSOD2 **(H)** cells, with or without DOX treatment, were induced to re-enter the cell cycle, harvested at 24 and 32 h, and subjected to flow cytometry with PI staining for cell cycle distribution analysis. **I, J** Quiescent DU145 shSOD2** (I)** and LNCaP shSOD2** (J)** cells, in the presence or absence of DOX, were treated with either DMSO or PTE (IC_50_ and IC_90_) for 48 h. Apoptosis was assessed by flow cytometry with Annexin V-APC/PI staining. 'Cont' refers to proliferative control cells, and 'Qsct' indicates quiescent cells. The data are presented as the means ± standard deviations from three individual experiments. ^**^P < 0.01, ^***^P < 0.001 *versus* the indicated groups.

To further explore the regulatory role of SOD2 in the reactivation of quiescent PCa cells, we established stable cell lines with doxycycline (DOX)-inducible SOD2 shRNA (DOX-shSOD2). PCa cells stably transfected with a parental empty vector (DOX-shCon) were used as controls. DOX treatment effectively reduced both SOD2 protein and mRNA expression levels in DU145 shSOD2 (Fig. 4A, C) and LNCaP shSOD2 cells (Fig. 4B, D). PCa cells, including nontransfected, DOX-shCon and DOX-shSOD2 cells, were treated with or without DOX on Day 5 of 7-day serum withdrawal and then reactivated into the cell cycle. SOD2 knockdown significantly increased the number of quiescent DU145 and LNCaP cells 48 h after serum reintroduction, indicating enhanced reproliferative capacity (Fig. 4E, F). In contrast, DOX treatment had no effect on the awakening of quiescent nontransfected or DOX-shCon PCa cells. Further analysis revealed that, compared with non-DOX-treated cells, DOX-treated shSOD2 PCa cells exhibited an accelerated shift from the G_0_/G_1_ to S and G_2_/M phases at 24 and 32 h (Fig. 4G, H). These findings further highlight the importance of SOD2 in quiescent PCa cell reactivation.

A rescue experiment was conducted to confirm the role of SOD2 in PTE-induced apoptosis during the awakening of quiescent PCa cells. DOX was administered to PCa shSOD2 cells 48 h before inducing cell cycle reentry. Subsequently, the cells were released from quiescence and exposed to PTE or control treatment for 48 h. SOD2 knockdown significantly reduced PTE-induced apoptosis in awakening quiescent DU145 and LNCaP cells. Notably, rescue percentages of 52.7% and 63.8% were achieved in PTE IC_50_- and IC_90_-treated DU145 shSOD2 cells, respectively (Fig. 4I, Fig. S5A). Similarly, rescue rates of 35.9% and 44.0% were observed in PTE IC_50_- and IC_90_-treated LNCaP shSOD2 cells, respectively (Fig. 4J, Fig. S5B). These results further confirm that PTE induces apoptosis during the awakening of quiescent PCa cells by increasing SOD2 expression.

### PTE upregulates SOD2 at the transcriptional level *via* C/EBP-β

**Figure 5:**
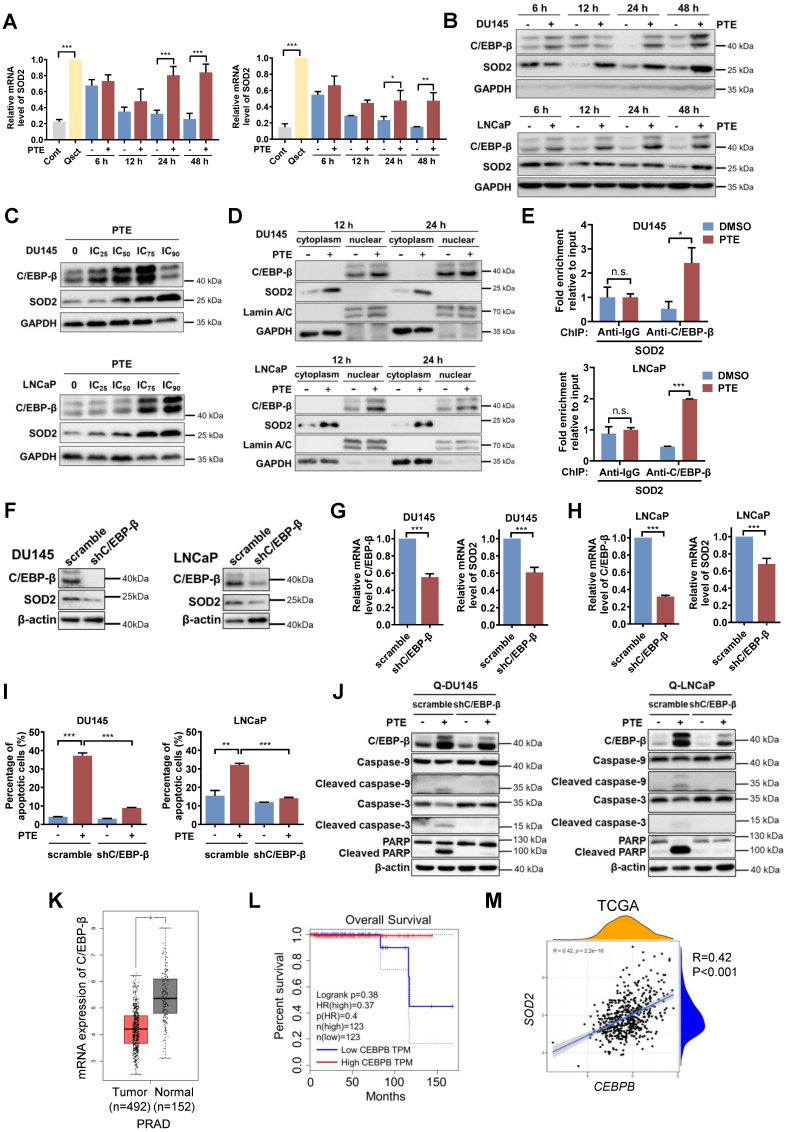
** PTE upregulates SOD2 at the transcriptional level *via* C/EBP-β. A** DU145 and LNCaP cells treated with PTE (IC_90_) during cell cycle reentry were analyzed for SOD2 mRNA levels by RT‒PCR, with values normalized to TBP. **B** Western blot analysis of C/EBP-β and SOD2 in awakening quiescent DU145 and LNCaP cells with PTE (IC_90_) treatment for the indicated intervals. GAPDH served as a loading control. **C** Western blotting was used to assess the expression of C/EBP-β and SOD2 in DU145 and LNCaP cells after PTE treatment at the indicated concentrations for 48 h. GAPDH was used as the loading control. **D** Protein expression levels of C/EBP-β and SOD2 in the nuclear and cytoplasmic extracts of awakening quiescent DU145 and LNCaP cells were analyzed using western blotting after PTE (IC_90_) treatment for 12 and 24 h. GAPDH and Lamin A/C were used as loading controls for cytoplasmic and nuclear extracts, respectively. **E** ChIP assay shows the enrichment of C/EBP-β on the SOD2 enhancer in awakening DU145 and LNCaP cells treated with either DMSO or PTE (IC_90_) for 24 h. **F-H** Western blotting **(F)** and RT‒PCR **(G, H)** analyses of C/EBP-β and SOD2 in quiescent DU145 and LNCaP cells transfected with the scramble and shC/EBP-β constructs. **I, J** Cell apoptosis was assessed by flow cytometry with Annexin V-FITC/PI staining **(I)**, and apoptosis-related protein levels were detected by western blotting **(J)** in quiescent DU145 and LNCaP cells with scramble control or shC/EBP-β knockdown after 48 h of PTE (IC_90_) treatment. **K** C/EBP-β expression levels in the prostate tissues of patients with PRAD (n=492) and normal controls (n=152) were analyzed using the GEPIA database.** L** Kaplan‒Meier survival curves from the GEPIA database showing the correlation between C/EBP-β expression and overall survival in patients with PRAD. **M** TCGA data revealed a positive association between *CEBPB* and *SOD2*. The data are presented as the means ± standard deviations from three individual experiments. n.s., not significant, ^*^P < 0.05, ^**^P < 0.01, ^***^P < 0.001 *versus* the indicated groups.

To further investigate the mechanism by which PTE upregulates SOD2, we first examined whether PTE treatment alters SOD2 transcription. The results demonstrated that SOD2 mRNA expression was greater in quiescent PCa cells than in proliferative cells. Upon release from quiescence, the mRNA expression of SOD2 decreased during the awakening of PCa cells, whereas PTE treatment significantly prevented this decrease in SOD2 mRNA expression (Fig. 5A), indicating PTE affects the transcription of SOD2. C/EBP-β, a transcription factor known to regulate SOD2 transcription 17, 27, was significantly upregulated by PTE treatment in a time- and dose-dependent manner after PCa cell reawakening (Fig. 5B, C). PTE markedly promoted the nuclear accumulation of C/EBP-β in awakening DU145 and LNCaP cells at 12 and 24 h after release from quiescence, accompanied by increased cytoplasmic SOD2 expression (Fig. 5D). To further confirm the transcriptional activity of C/EBP-β in regulating SOD2 expression after PTE treatment, ChIP analysis was performed. Compared with the control group, antibody against C/EBP-β significantly increased the interaction with *SOD2* intronic enhancer following PTE treatment during the reactivation of quiescent PCa cells (Fig. 5E).

To determine whether C/EBP-β plays a crucial role in PTE-induced apoptosis in awakening PCa cells, we established stable C/EBP-β knockdown DU145 and LNCaP cell lines using lentiviral transfection of shC/EBP-β plasmids, with a nonsense plasmid (scramble) used as a control. Knockdown of C/EBP-β in quiescent PCa cells led to reductions in both C/EBP-β and SOD2 protein levels (Fig. 5F) as well as in C/EBP-β mRNA levels (Fig. 5G, H). C/EBP-β knockdown significantly reduced PTE-induced apoptosis in awakening quiescent PCa cells compared with control cells (Fig. 5I; Fig. S6C, D). Consistently, the PTE-induced increases in the expression of cleaved caspase-3, caspase-9 and PARP were diminished by C/EBP-β knockdown (Fig. 5J). Taken together, PTE upregulates SOD2 and induces apoptosis in awakening PCa cells, at least partially by enhancing the transcriptional activity of C/EBP-β on SOD2.

To further evaluate the clinical relevance of C/EBP-β in human PCa tissue, we examined its expression using the publicly available GEPIA database, which includes 492 samples from patients with PRAD and 152 normal human prostate samples (Fig. 5K). The results revealed that C/EBP-β mRNA expression was significantly lower in PRAD tissues than in normal prostate tissues. Kaplan‒Meier survival analysis revealed that reduced C/EBP-β in PRAD expression correlated with reduced overall survival in patients with PCa (Fig. 5L). Additionally, analysis of the TCGA database revealed a positive correlation between *CEBPB* and *SOD2* (Fig. 5M), indicating that *CEBPB* tightly regulates *SOD2* expression. Together, these data indicate that C/EBP-β plays a critical role in PTE-induced eradication of awakening PCa cells from quiescence by upregulating SOD2, highlighting its therapeutic potential for preventing PCa recurrence.

### PTE suppresses the growth of recurrent tumors derived from quiescent PCa cells* in vivo*

**Figure 6:**
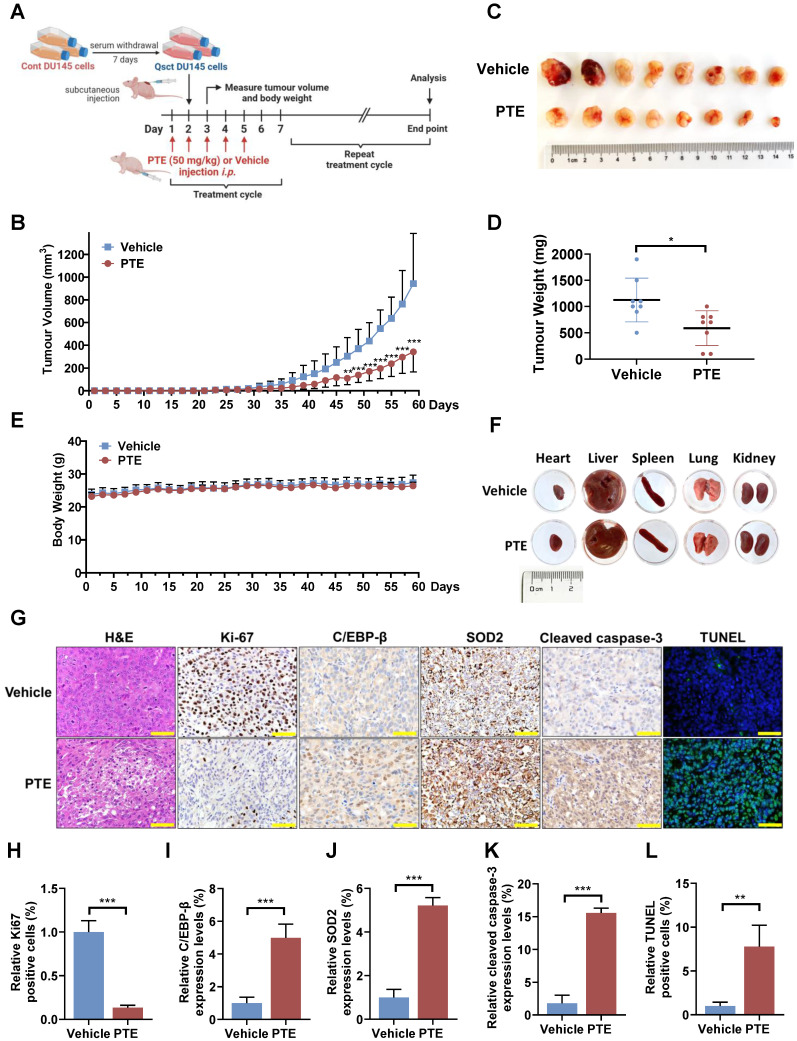
** PTE suppresses the regrowth of quiescent PCa tumors *in vivo*. A** Schematic diagram of quiescent PCa tumor regrowth in a PTE-treated xenograft model.* i.p.*, intraperitoneally. **B-E** Tumor volumes (**B**) and mouse body weights (**E**) were measured every other day, and the mice were euthanized when the tumor volume reached 1000 mm^3^. On Day 59, the tumors were excised, photographed (**C**) and weighed (**D**). **F** Representative heart, liver, spleen, lung and kidney morphologies in mice treated with vehicle control or PTE (50 mg/kg) on the day of sacrifice. **G-L** Paraffin-embedded tumor tissues were subjected to H&E staining, TUNEL, and immunostaining with antibodies against Ki-67, C/EBP-β, SOD2 and cleaved caspase-3. Three randomly selected fields per section (n = 3 per group) were quantitatively analyzed using ImageJ software. Representative images (**G**) and quantification data (**H-L**) are presented. Scale bar: 50 μm. The data are presented as the means ± standard deviations. ^*^P < 0.05, ^**^P < 0.01, ^***^P < 0.001 *versus* the control group or indicated group.

The reactivation of quiescent cancer cells is widely recognized as a key contributor to cancer recurrence 28, 29. To further investigate the potential of PTE in preventing the reawakening of quiescent PCa cells *in vivo*, quiescent DU145 cells were subcutaneously implanted in mice to allow for recurrent tumor growth. The mice were administered PTE (50 mg/kg) or vehicle intraperitoneally starting the day before cell implantation and continuing weekly for five consecutive days over two months (Fig. 6A). In the vehicle-treated group, measurable tumors (100 mm³) began to appear by Day 39, with tumor growth progressing until the ethical endpoint (1000 mm³) was reached on Day 59 (Fig. 6B). In contrast, the PTE-treated group exhibited a significant delay in tumor regrowth, with measurable tumors appearing on Day 49. Compared with vehicle-treated mice, PTE-treated mice developed smaller tumors, with an average reduction in tumor weight of 65% after 59 days (Fig. 6C, D). Moreover, no significant changes in body weight or the gross anatomy of major organs were observed during PTE treatment (Fig. 6E, F). Histological analysis of tumors from PTE-treated mice revealed decreased cell density (H&E staining), a decreased number of Ki-67-positive cells, elevated protein levels of C/EBP-β, SOD2 and cleaved caspase-3, and an increased number of TUNEL-positive cells (Fig. 6G-L).

## Discussion

Androgen signaling, predominantly mediated through the androgen receptor (AR), is essential for the initiation and progression of PCa. Androgen-deprivation therapy (ADT), which inhibits androgen production or suppresses AR function, remains the first-line treatment for advanced and metastatic PCa 30, 31. Although ADT initially results in disease regression, most patients experience only a transient response and ultimately develop to castration-resistant prostate cancer (CRPC) 32, 33. Following treatments such as prostatectomy, radiotherapy, or ADT, a dormant phase may occur during which residual QCCs persist. These cells can later reactivate, ultimately leading to tumor recurrence 34. PCa thus serves as an ideal model for investigating tumor dormancy, given that many patients often remain disease-free for over a decade post-prostatectomy before developing biochemical recurrence or metastasis 35, 36. Therefore, strategies aimed at preventing the reactivation of QCCs hold significant promise for reducing recurrence and improving long-term patient outcomes. In our study, we demonstrate that SOD2 overexpression is critical for preventing QCC reactivation by inducing apoptosis, suppressing recurrent tumor growth, and prolonging survival in preclinical models. Moreover, our findings report for the first time that PTE effectively blocks the reawakening of QCCs through apoptosis induction, highlighting its potential to prevent PCa recurrence.

PTE, a natural compound found in herbal medicines and edible fruits such as blueberries and grapes, is typically produced through both biosynthesis and chemical synthesis. PTE has exhibited no significant toxicity in both preclinical and clinical trials 21, 37, 38, making it a promising and cost-effective drug candidate for long-term therapy. Notably, our results revealed a robust induction of apoptosis by PTE in both LNCaP (AR-positive) and DU145 (AR-negative) cells during awakening. This finding holds significant clinical implications for treating ADT-resistant PCa phenotypes such as CRPC. Developing clinically pertinent patient-derived xenograft (PDX) models, using serum prostate specific antigen (PSA) levels and tumor volume as key indicators, can better mimic ADT-induced dormancy in the clinic 32, 39, 40. In parallel, it is imperative to establish* in vitro* models using media supplemented with charcoal-stripped FBS to simulate prolonged androgen-deprived conditions 41, 42, which are critical for studying the dormancy and subsequent reactivation of PCa cells. These experimental platforms can facilitate detailed mechanistic investigations into the role of PTE in androgen deprivation-induced PCa dormancy. Moreover, future research should elucidate whether AR plays a pivotal role in mediating PTE-induced apoptosis in quiescent PCa cells and explore the underlying molecular mechanisms. Furthermore, evaluating the potential synergistic effects of combining PTE with standard therapies in localized or advanced PCa may offer novel strategies for enhancing clinical outcomes.

In our study, SOD2 knockdown reduced PTE-induced apoptosis in awakening quiescent PCa cells, with rescue percentages of 63.8% and 44.0% observed in PTE IC_90_-treated DU145 and LNCaP shSOD2 cells, respectively (Fig. 4I, J). This partial rescue suggests that additional mechanisms may contribute to PTE-induced apoptosis. Studies in proliferating PCa cells have demonstrated that PTE triggers apoptosis through multiple pathways, including enhanced ROS production, downregulation of Akt and Bcl-2, activation of mitochondrial apoptotic cascades, and AMPK activation 43, 44. Moreover, PTE has been reported to induce apoptosis in non‑small‑cell lung cancer cells by suppressing cyclooxygenase-2 expression and in gastric cancer cells by downregulating mitochondria‑related genes 45. This downregulation leads to mitochondrial iron (II) accumulation and ROS‑mediated activation of HIF‑1α, which subsequently triggers endoplasmic reticulum stress and apoptosis 46, 47. Further investigation is needed to determine their relative contributions in PTE-induced apoptosis in quiescent PCa cells.

Besides, genetic overexpression of SOD2 demonstrated more sustained tumor suppression than PTE, likely due to the inherent limitations of small-molecule therapeutics. PTE is rapidly metabolized by hepatic cytochrome P450 enzymes (CYP1A2, CYP2C9, and CYP3A4) 48, and the tumor microenvironment may further hinder its penetration and activity. In its native form, PTE may not fully meet the pharmacological demands in humans due to limited bioavailability; therefore, structural modifications (such as alterations to its carbon-carbon double bonds or benzene ring) have been developed to improve its chemopreventive and therapeutic potential 49. Future strategies, including prodrug modifications, nanoparticle-based delivery, or alternative formulations, could enhance its clinical bioavailability by improving water solubility, enabling controlled drug release, and increasing stability under physiological conditions 48. For its clinical advancement, future research should include comprehensive* in vivo* studies, such as PCa PDX models, to assess long-term efficacy and recurrence prevention. Detailed pharmacokinetic and toxicological evaluations in PCa animal models are also necessary to establish its safety profile. Additionally, Phase I/II clinical trials will be essential to confirm tolerability and to provide preliminary efficacy data in PCa patients.

Recent toxicity studies in both animals and humans support the safety of PTE at therapeutic doses. For example, dietary intake of up to 3000 mg/kg/day in Swiss mice increased red blood cell counts without significant alterations in serum proteins, electrolytes, or liver/kidney enzymes, and histopathological assessments of major organs revealed no toxicity-related changes 50. Moreover, a clinical study in adults confirmed that daily doses of up to 250 mg of PTE are safe, with no significant adverse effects on liver, kidney, or glucose markers compared to placebo 38. Therefore, although the applications of PTE in cancer therapy are still in its early stages, it represents a promising and effective agent that may enhance oncotherapy outcomes and serve as a valuable adjuvant therapy following chemotherapy.

Preventing cancer recurrence poses a significant therapeutic challenge, as the underlying mechanisms are complex and not yet fully understood. Our data reveal that SOD2 is overexpressed in quiescent PCa cells, which is in line with observations in other cancer types. For example, in head and neck squamous cell carcinoma, quiescent Cal27 and FaDu cells exhibited a three- to tenfold increase in SOD2 mRNA compared to proliferating cells 51. Similarly, SOD2 was significantly upregulated by the kinase Mirk/Dyrk1B in quiescent pancreatic cancer cells 52. Our findings demonstrate for the first time that overexpression of SOD2 is critical in preventing the reactivation of QCCs by inducing apoptosis, suppressing the growth of recurrent tumours from quiescent PCa cells, and prolonging mice survival. Although SOD2 is conventionally characterized as a protective antioxidant, catalyzing the conversion of mitochondrial superoxide radicals into hydrogen peroxide (H₂O₂) and oxygen (O₂) 53, emerging evidence indicates that its overexpression can also exert tumor-suppressive effects 54-58. Moreover, the H₂O₂ generated by SOD2 can either be metabolized to water or participate in the Fenton reaction in the presence of iron (Fe^2+^ + H_2_O_2_ → Fe^3+^+ ·OH + OH^-^), generating damaging hydroxyl radicals, thereby contributing to cell death 59.

Cancer dormancy is frequently characterized by substantial alterations in mitochondrial respiration and metabolic reprogramming 60. Consequently, quiescent cells are less susceptible to apoptosis, as they typically employ differential anti-apoptotic mechanisms to ensure their survival 1, 61. QCCs often display variable oxidative phosphorylation (OXPHOS) activity—enhanced OXPHOS can optimize ATP production under low proliferative demand, while reduced metabolic activity and OXPHOS may be linked to reduced proliferative signaling in QCCs 62-64. Moreover, quiescent cancer stem-like cells are generally characterized by reduced metabolic activity, which minimizes ROS production 65, 66. This distinct metabolic phenotype not only enables these cells to evade cytotoxic agents that target rapidly dividing cells but also is integral to their long-term survival 67, 68. In our study, PTE treatment paradoxically increased mitochondrial ROS levels during cell cycle reentry; however, the mitochondrial ROS scavenger, mitoTEMPO, failed to mitigate PTE-induced apoptosis (Fig. S3). This suggests that the pro-apoptotic effects of PTE-mediated SOD2 upregulation are not solely due to changes in mitochondrial ROS levels. Instead, PTE may interfere with mitochondrial complex activities, alter SOD2 catalytic efficiency through acetylation of key lysine residues (K68 and K122) 69, 70, or promote the accumulation of an iron-bound SOD2 form (FeSOD2) with peroxidase activity 53, 71. Additionally, the interplay between SOD2 and other antioxidant systems, such as catalase, glutathione peroxidase, and peroxiredoxin, adds further complexity to the regulation of ROS 72, 73.

Our study demonstrated that C/EBP-β significantly enhanced the association with *SOD2* intronic enhancer following PTE treatment during the reactivation of quiescent PCa cells, thereby promoting SOD2 transcription. Similar mechanisms have been reported in previous studies. For example, in human endometrial stromal cells, cAMP-induced SOD2 expression is dependent on C/EBP-β binding with the enhancer region, and the full-length LAP isoform of C/EBP-β was identified as a critical activator of SOD2 transcription 27, 74. Further investigation through site-directed mutagenesis of these binding sites could help delineate the specific role of C/EBP-β in PTE-treated awakening quiescent PCa cells. Notably, knockdown of C/EBP-β in quiescent PCa cells partially reduced SOD2 expression, suggesting that additional regulatory signaling pathways may be involved. It has been reported that NF‑κB can cooperate with C/EBP-β to regulate murine SOD2 *via* a complex intronic enhancer in response to TNF-α and IL-1β 75. The regulatory mechanisms in quiescent cells warrant further investigation. Besides, C/EBP-β knockdown did not impair the ability of DU145 and LNCaP cells to enter or maintain quiescence (Fig. S6A, B), but it accelerated cell cycle reentry (Fig. S6E, F). Clinical data further corroborate these findings, as higher levels of C/EBP-β correlate with increased SOD2 expression and improved prognosis in PCa patients. Collectively, these results suggest that targeting C/EBP-β-SOD2 axis may be an effective strategy for eliminating awakening QCCs and preventing cancer recurrence.

In conclusion, our study demonstrates that PTE, a natural, low-toxicity compound with, effectively induces apoptosis in awakening quiescent PCa cells, suppresses recurrent tumor growth, and prolongs survival. The C/EBP-β-SOD2 signaling pathway is pivotal in PTE-mediated eradication of quiescent PCa cells during reactivation and represents a promising therapeutic target for preventing PCa recurrence.

## Materials and methods

### Chemicals and reagents

PTE (T2888, 99.71% purity) was obtained from TargetMol (Boston, MA, USA). It was dissolved in dimethyl sulfoxide (DMSO; 543900, Sigma‒Aldrich, Carlsbad, CA, USA), and stored at -20 °C. Z-VAD-FMK (C1202), RIPA lysis buffer (P0013C), crystal violet staining solution (C0121) and protease inhibitor cocktail (P1005) were obtained from the Beyotime Institute of Biotechnology (Shanghai, China). RNAiso Plus (TRIzol) and the PrimeScript RT Reagent Kit (RR037A) were purchased from Takara Biotechnology (Shiga, Japan). Additional reagents included SYBR Green Real-time PCR Master Mix (QPK-201, Toyobo Life Science, Osaka, Japan), propidium iodide (PI, P4170, Sigma‒Aldrich), SYBR Green (S-7563, Life Technologies, Carlsbad, CA, USA), an Annexin V-FITC/PI Apoptosis Kit (Meilun Biotechnology, Dalian, China) and an Annexin V-APC/PI Apoptosis Kit from Multi sciences Biotech (Hangzhou, China).

### Cell culture

Human prostate cancer cell lines DU145 and LNCaP, along with the human embryonic kidney cell line HEK‑293T, were obtained from the American Type Culture Collection (Manassas, VA, USA). The PCa cell lines and HEK-293T cells were cultured in complete RPMI 1640 medium (MB4374) or DMEM (MA0212) from Meilun Biotechnology, respectively, with 10% fetal bovine serum (04-001-1 ACS, Biological Industries, Israel). The cells were maintained in a humidified atmosphere in a humidified atmosphere containing 5% CO₂ and 95% air. Experimental quiescence of PCa cells was achieved following established protocols 5. Briefly, DU145 and LNCaP cells were cultured to 80% confluence, followed by serum deprivation for an additional 7 days. To induce cell cycle reentry, the cells were replated in the presence of serum. The cell culture materials were procured from NEST Biotechnology Co., Ltd. (Wuxi, China).

### Western blotting

Cell pellets were lysed using ice-cold RIPA buffer supplemented with a protease inhibitor cocktail. Protein quantification, electrophoresis, transfer and immunoblotting were carried out following previously described methods 76. The membranes were incubated with primary antibodies: anti-SOD2 (sc-137254), anti-p27 (sc-528) and anti-C/EBP-β (sc-7962) from Santa Cruz Biotechnology (Dallas, CA, USA); anti-caspase-3 (9662), anti-poly ADP-ribose polymerase (PARP, 9542), anti-caspase-9 (9502) and anti-phosphorylated retinoblastoma (phospho-Rb, Ser807/811, 9308) from Cell Signaling Technology (Danvers, MA, USA); β-actin (66009-1-lg) and GAPDH (60004-1-Ig) from Proteintech (Wuhan, China); and Lamin A/C (ab108595) from Abcam (Cambridge, United Kingdom).

For the subcellular fractionation assay, cytoplasmic and nuclear proteins were isolated using a nuclear and cytoplasmic protein extraction kit (Beyotime Biotechnology) and subsequently analyzed by western blotting. Lamin A/C and GAPDH served as controls for the nuclear and cytoplasmic fractions, respectively.

### Real-time polymerase chain reaction (RT‒PCR)

Total RNA was extracted from cells using TRIzol reagent, and then quantified using a NanoDrop spectrophotometer (DeNovix, Wilmington, DE, USA); reverse transcription was conducted with a PrimeScript RT Reagent Kit. RT‒PCR was performed using the SYBR Green Real-time PCR Master Mix on a StepOnePlus Real-Time PCR System (Applied Biosystems, Carlsbad, CA, USA). The experimental protocol was consistent with a previous description, and normalization was performed relative to the expression level of TATA box-binding protein (TBP) 23. Primer sequences were as follows: 5′-GAAC CACG GCAC TGAT TTTC-3′ (forward) and 5′-CCCC ACCA TGTT CTGA ATCT-3′ (reverse) for TBP; 5′-ATTT GTAA GTGT CCCC GTTC C-3′ (forward) and 5′-GTGG TGGT CATA TCAA TCAT AGC-3′ (reverse) for SOD2; 5'-CCCG CCCG TGGT GTTA TTTA-3' (forward) and 5'-CACG CGTT CAGC CATG TTTA-3' (reverse) for CEBPB. All expression levels were quantified using StepOne Software version 2.3 and analyzed with Microsoft Excel software.

### Establishment of doxycycline (DOX)-inducible stable SOD2 shRNA and stable C/EBP-β shRNA cell lines

A lentiviral GFP-IRES-DOX-inducible FH1tUTG shRNA expression construct, generously provided by Dr. Marco Herold, has been previously characterized 59. The SOD2 shRNA sequence, GGTG GTCA TATC AATC ATAG CTTC AAGA GAGC TATG ATTG ATAT GACC ACC, was introduced into the DOX-inducible GFP-IRES-shRNA FH1tUTG construct, whereas the unaltered FH1tUTG served as the empty vector (EV) control. The PGMLV-hU6-MCS-CMV-Puro-WPRE construct, procured from Genomeditech (Shanghai, China), was used to silence C/EBP-β. Lentiviral preparation was conducted following the established protocol 39. The DOX-inducible EV shRNA, DOX-inducible SOD2 shRNA, scramble shRNA or C/EBP-β shRNA constructs were introduced into HEK-293T cells *via* transfection using a Lenti-Pac^TM^ HIV Expression Packaging Kit (GeneCopoeia, Changzhou, China). Viral supernatants were collected and used to infect DU145 and LNCaP cells in serum‑free medium. Cells expressing high levels of GFP were selected with 0.2 mg/ml puromycin (REVG1001; GeneChem, Shanghai, China). For *in vitro* induction of Dox-inducible shRNA expression, the cells were exposed to 4 µg/ml doxycycline (DOX, ST039A; Beyotime Institute of Biotechnology, Shanghai, China) for 48 h.

### Establishment of stable SOD2-overexpressing PCa cell lines

PGMLV-CMV-Empty Vector-3×Flag-EF1-mCherry-T2A-Puromycin (EV) and PGMLV-CMV-SOD2-3×Flag-EF1-mCherry-T2A-Puromycin (SOD2) expression constructs were procured from Genomeditech (Shanghai, China) and subsequently transfected into HEK-293T cells using a Lenti-Pac^TM^ HIV Expression Packaging Kit (GeneCopoeia, Changzhou, China). The viral media were then harvested and stored at -80 °C. DU145 and LNCaP cells were infected with the lentiviral particles in serum-free growth medium. The culture medium was replaced with complete RPMI 1640 medium, followed by incubation and subsequent selection with 0.2 mg/ml puromycin.

### Cell cycle analysis

Quiescent PCa cells or PCa cells transfected with various plasmids were stimulated to re-enter the cell cycle with or without PTE treatment for different durations. Cells were collected, fixed overnight in ice‑cold 70% ethanol prepared in phosphate‑buffered saline (PBS) and subsequently stored at 4 °C. Flow cytometry with propidium iodide (PI) staining was subsequently conducted according to previously established protocols 23. The distribution of the cell cycle phases was analyzed to determine the proportion of cells in each phase using FlowJo software (version 7.6.1).

### Cell viability assay

Cell viability was evaluated using a Trypan Blue Staining Assay Kit (C0011) from the Beyotime Institute of Biotechnology. Quiescent DU145 and LNCaP cells, along with DOX-inducible shCon/shSOD2 DU145 and LNCaP cells (2 × 10^4^ cells/well), with or without DOX treatment, were seeded into 24-well plates and maintained in complete medium to induce proliferation for 48, 72, 96 and 120 h. The cells were collected at the indicated intervals, and 20 µl of the cell suspension was combined with an equal volume of Trypan blue staining solution. The viable cells were then analyzed and counted using a Countstar instrument (Alit Biotech, Shanghai, China).

A PI exclusion assay was also employed to assess cell viability. Quiescent EV and SOD2-overexpressing DU145 and LNCaP cells were plated in 6 cm dishes and activated to proliferate for 8, 16 and 24 h. The cells were collected, suspended in PBS supplemented with 100 µg/ml RNase and stained with PI solution (20 µg/ml) for 5 min. Flow cytometry (FACSCalibur, BD Biosciences) was utilized to determine the percentage of viable and dead cells, with analysis by FlowJo software (version 7.6.1).

### Mitochondrial membrane potential detection

Mitochondrial membrane potential was measured with a JC-1 staining assay kit (C2006, Beyotime), as previously described 77. Quiescent EV- and SOD2-overexpressing DU145 and LNCaP cells were plated in 6 cm dishes and simulated to re-enter the cell cycle in the presence or absence of Z-VAD-FMK (50 μM) for 12 and 24 h. Subsequently, they were harvested and stained with JC-1 at 37 °C for 20 min, rinsed and analyzed by flow cytometry (FACSCalibur, BD Biosciences).

### SYBR Green assay

Quiescent DU145 (8 × 10^3^ cells per well) and LNCaP (1 × 10^4^ cells per well) cells were stimulated to resume the cell cycle by culturing them in complete medium in 96-well plates, supplemented with specified concentrations of PTE for 72 h. After removing the culture medium, the cells were stored at -80 °C. An equivalent number of synchronized cells was retained as a baseline condition and stored at -80 °C for comparison. The SYBR Green assay was performed following a previously established protocol 22. DNA quantification data were analyzed using Microsoft Excel, and the inhibitory concentrations (ICs) for the cells [25% (IC_25_), 50% (IC_50_), 75% (IC_75_) and 90% (IC_90_)] were calculated using the following formula: [1 - (DNA quantity in the PTE group)/(DNA quantity in the control group)] × 100%.

### Colony formation assay

Quiescent DU145 (1500 cells/well) and LNCaP (800 cells/well) cells were stimulated to re-enter the cell cycle by culturing them in complete medium containing either DMSO or PTE (at IC_25_, IC_50_ and IC_75_ concentrations) in 6-well plates for 24, 48 and 72 h. Afterwards, the culture medium was replaced with PTE-free complete medium, and the cells were incubated for an additional 14 days to allow colony formation. Colonies were fixed in 95% ethanol, stained with a 1% crystal violet solution in PBS and then imaged. Colonies containing more than 50 cells were counted as clones.

### Annexin V/PI double-staining assay

Cell cycle reentry was induced in quiescent PCa cells by seeding them into 6 cm dishes (1 × 10^6^ cells) containing complete medium with either DMSO, PTE (at IC_50_ and IC_90_ concentrations) or Z-VAD-FMK (50 μM) for 24 and 48 h. The cells were then harvested, washed and stained with either an Annexin V-FITC/PI or an Annexin V-APC/PI Apoptosis Kit according to the manufacturer's protocols, with apoptotic cells quantified by flow cytometry (FACSCalibur, BD Biosciences), with Annexin V-positive cells considered indicative of apoptosis.

### Chromatin immunoprecipitation (ChIP) assay

ChIP assay was conducted using a ChIP Assay Kit (P2087, Beyotime) following the manufacturer's protocol. Cells were fixed with 1% formaldehyde, and nuclei were subsequently extracted. The chromatin/DNA complex was fragmented using sonication. The lysates were clarified and incubated overnight at 4 °C with protein A+G agarose beads coupled with an anti-C/EBP-β antibody (23431-1-AP, Proteintech). Normal rabbit IgG (A7016, Beyotime) served as the negative control. The DNA was eluted and analyzed by qPCR. The primer sequences for the SOD2 enhancer region were as follows: 5′- AAGT GTGG TATT TTAG CATA GTTG TGTA-3′ (forward) and 5′- AGAG GAAA GTTG TCAG ATGT CACC-3′ (reverse).

### Public dataset-based bioinformatic analysis

The Gene Expression Profiling Integrative Analysis (GEPIA) database was used to analyze expression profiles from The Cancer Genome Atlas database and the Genotype-Tissue Expression project. GEPIA also facilitated differential expression analysis of C/EBP-β in prostate adenocarcinoma (PRAD) tumor tissue *versus* normal tissue. The correlation between C/EBP-β expression and overall survival in patients with PRAD was analyzed using GEPIA with a Cox model 78. Data were downloaded from the TCGA-PRAD database (https://portal.gdc.cancer.gov/), and Pearson's correlation between CEBPB and SOD2 expression levels was analyzed using the "ggpubr" R package. Correlation plots were generated to visualize the results based on the TCGA expression data.

### Establishment of tumor xenografts in nude mice

All animal experiments were approved by the Shanghai University of Traditional Chinese Medicine Committee for the Use of Live Animals for Teaching and Research (approval numbers PZSHUTCM200717024 and PZSHUTCM201225005), and conducted in accordance with institutional guidelines. Four-week-old male BALB/c nude mice were obtained from the Experimental Animal Center of the Chinese Academy of Sciences (Shanghai, China) and housed in a sterile, pathogen-free environment. The mice were randomly divided into two groups of nine and subcutaneously injected in the right flanks with 4.3 × 10^6^ quiescent EV DU145 or SOD2-overexpressing DU145 cells. Tumor volume was measured every other day using a digital caliper by recording the length (L), width (W) and height (H) and was calculated using the following formula: 3.14 × L × W × H/6). Mice were euthanized when tumor volumes reached 1000 mm³, and tumors were excised, weighed and photographed.

For the PTE treatment study, four-week-old male BALB/c nude mice were subcutaneously injected with 3.6 × 10^6^ quiescent DU145 cells in the right flank and randomly divided into two groups (n = 8 per group). PTE (50 mg/kg) and a vehicle control (10% Cremophor EL, 10% absolute ethanol and 5% glucose in saline) were administered intraperitoneally one day before tumor cell implantation. PTE and the vehicle control were then administered from Days 1 to 5 of each week for two months, with tumor volume and body weight measured every other day. After 61 days, the mice were euthanized, and the tumors were excised, weighed and photographed.

### Immunohistochemistry

Immediately after euthanasia, tumors were harvested and fixed in 4% neutral buffered paraformaldehyde. The samples were then embedded in paraffin and sectioned into 5 µm thick slices. Subsequently, the samples underwent hematoxylin and eosin (H&E) staining as well as TUNEL staining (MA0223, Meilun Biotechnology, Dalian, China), and staining with antibodies against Ki-67 (ab16667, Abcam), SOD2 (ab68155, Abcam), cleaved caspase-3 (#9661, Cell Signaling Technology) and C/EBP-β (23431-1-AP, Proteintech). The sections were prepared for histological analysis by mounting them with DPX. Three views were randomly selected from each section (n=3 for each group) and the area of each section was quantitatively measured using ImageJ software. The IHC quantification was conducted using ImageJ following a standardized protocol: each IHC-stained image was converted to an RGB stack, a threshold was applied to isolate the positive staining, and measurements were set to obtain the “% Area” values.

### Statistical analysis

Statistical analysis was conducted based on experiments independently repeated at least three times, and data are presented as the mean ± standard deviations from three biological replicates. Data analysis was performed using GraphPad Prism 10 software and SPSS (version 23.0) statistical software. Comparisons between two groups were performed using Student's two-tailed *t* test, whereas comparisons among more than two groups were conducted using ANOVA with Fisher's least significant difference (LSD) multiple comparisons test. Survival analyses were conducted using the Kaplan‒Meier method in GraphPad Prism 10 software, and comparisons between survival curves were assessed using the two-sided log-rank test. The levels of statistical significance were denoted as ^*^P < 0.05, ^**^P < 0.01 and ^***^P < 0.001.

## Supplementary Material

Supplementary figures.

## Figures and Tables

**Table 1:** IC values of PTE in quiescent PCa cells

IC_%_	DU145 (μM)	LNCaP (μM)
IC_25_	17.51 ± 0.51	19.73 ± 1.21
IC_50_	34.45 ± 0.74	34.31 ± 1.44
IC_75_	67.79 ± 2.42	59.73 ± 2.43
IC_90_	133.47 ± 7.58	104.04 ± 5.98

The IC values of PTE were calculated based on the results of the SYBR Green assay (Fig. 1A, B) using GraphPad Prism 10. The IC values of each cell line are shown as the mean ± standard errors.

## Abbreviations

ADT: androgen-deprivation therapy
AMPK: AMP-activated protein kinase
AR: androgen receptor
ATP: adenosine triphosphate
C/EBP-β: CCAAT/enhancer-binding protein beta
Cont: control cells (proliferative cells)
CRPC: castration-resistant prostate cancer
ChIP: chromatin immunoprecipitation
DMSO: dimethyl sulfoxide
DOX: doxycycline
EV: empty vector
FBXW7: F-box and WD repeat domain containing 7
FBS: fetal bovine serum
FITC: fluorescein isothiocyanate
GEPIA: gene expression profiling integrative analysis
H₂O₂: hydrogen peroxide
H&E: hematoxylin and eosin
IC: inhibitory concentration
Mirk/Dyrk1B: minibrain-related kinase/dual-specificity tyrosine phosphorylation-regulated kinase 1B
NEPC: neuroendocrine prostate cancer
O_2_: oxygen
OXPHOS: oxidative phosphorylation
PARP: poly ADP-ribose polymerase
PBS: phosphate-buffered saline
PCa: prostate cancer
PI: propidium iodide
PDX: patient-derived xenograft
PRAD: prostate adenocarcinoma
PSA: prostate specific antigen
PTE: pterostilbene
QCCs: quiescent cancer cells
Qsct: quiescent cells
Rb: retinoblastoma
ROS: reactive oxygen species
RT‒PCR: real-time polymerase chain reaction
SOD2: superoxide dismutase 2
TCGA: The Cancer Genome Atlas
TBP: TATA box-binding protein
