# Supplementary material for: Pterostilbene Induces Apoptosis in Awakening Quiescent Prostate Cancer Cells by Upregulating C/EBP-β-Mediated SOD2 Transcription
Source: Int J Biol Sci. 2025 May 7;21(8):3379–96. doi: 10.7150/ijbs.106219 (PMC12160548; doi:10.7150/ijbs.106219)
Supplement: Supplementary file 1 — Supplementary figures. [file ijbsv21p3379s1.pdf]

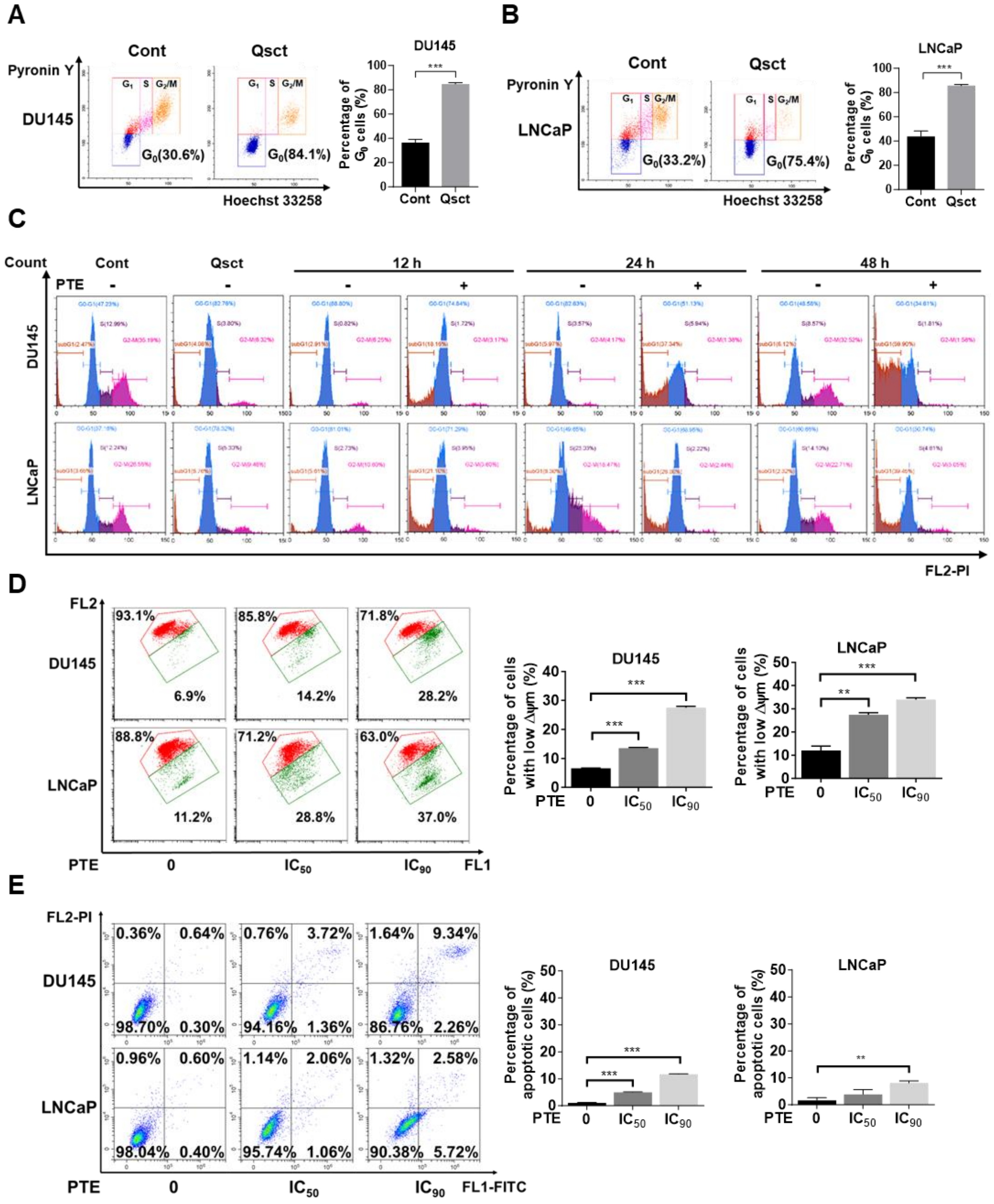

Figure S1. PTE induces significant cell death in awakening quiescent PCa cells compared to proliferative cells. A, B Representative flow cytometry plots of DU145 **(A)** and LNCaP **(B)** cells stained with Hoechst 33258 and Pyronin Y (left), with the quantification on the right. **C** Quiescent DU145 and LNCaP cells were induced to re-enter the cell cycle with DMSO or PTE (IC_{90}) treatment. The cells were harvested at the indicated intervals and analyzed for cell cycle distribution using PI-stained flow cytometry. **D** DU145 and LNCaP cells were released from quiescence and treated with

PTE (IC_{50} and IC_{90}) for 24 h, followed by JC-1-stained flow cytometry to detect mitochondrial membrane potential. **E** Proliferative DU145 and LNCaP cells were treated with either DMSO or PTE (IC_{50} and IC_{90}) for 48 h, followed by Annexin V-FITC/PI-stained flow cytometry to assess apoptosis. Representative images (left) and quantification data (right) of apoptotic cells are shown. 'Cont' refers to proliferative control cells, and 'Qsct' indicates quiescent cells. Data are presented as mean ± standard deviation. $^{**}P < 0.01$, $^{***}P < 0.001$ *versus* the indicated group.

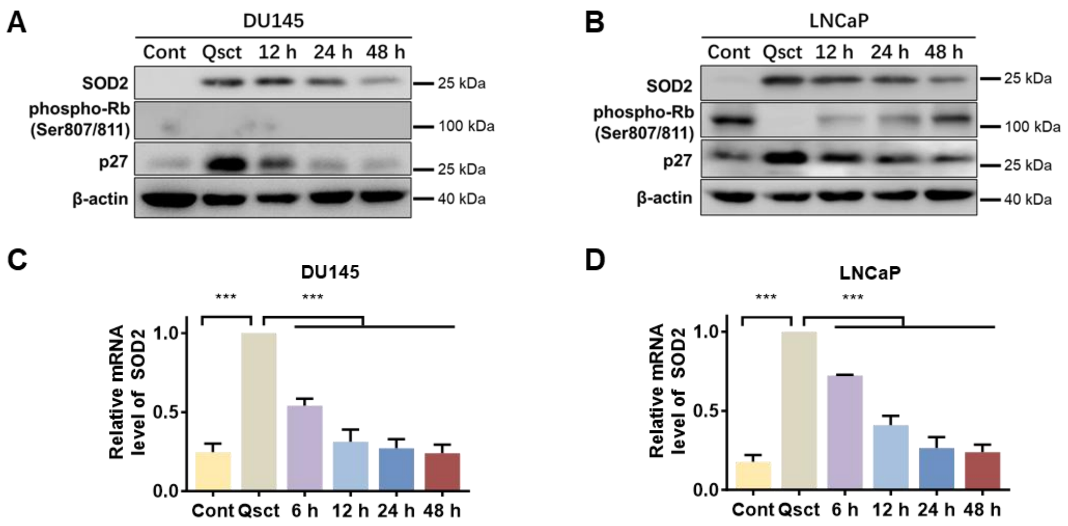

Figure S2. SOD2 is overexpressed in quiescent PCa cells and decreases during awakening. A, B Protein levels of SOD2, phospho-Rb (Ser807/811) and p27 were detected in proliferative, quiescent, and cell cycle re-entry phases (12–48 h) in DU145 (**A**) and LNCaP (**B**) cells. β-actin served as a loading control. **C, D** Relative SOD2 mRNA levels in quiescent DU145 (**C**) and LNCaP (**D**) cells during cell cycle re-entry were measured at the indicated intervals and normalized to TBP. 'Cont' refers to proliferative control cells, and 'Qsct' indicates quiescent cells. Data are presented as

mean ± standard deviation from three individual experiments. $^{***}P < 0.001$ *versus* the indicated groups.

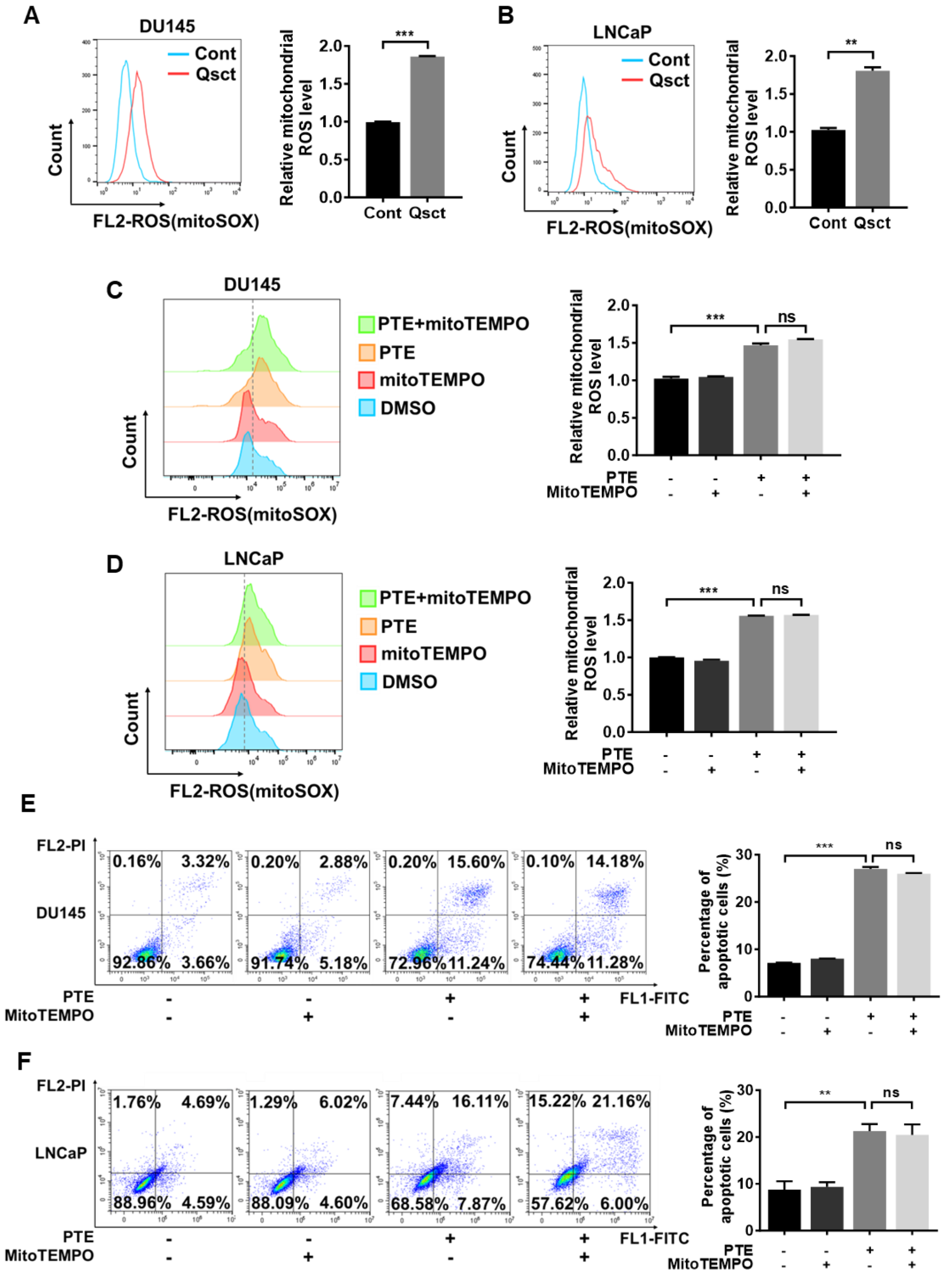

Figure S3. PTE further elevates mitochondrial ROS levels in quiescent PCa cells.

A, B Mitochondrial ROS levels in proliferative and quiescent DU145 (**A**) and LNCaP

(**B**) cells were measured using mitoSOX probes *via* flow cytometry. **C, D** Mitochondrial ROS levels in awakening quiescent DU145 (**C**) and LNCaP (**D**) cells were detected following co-treatment with PTE (IC_{90}) and mitoTEMPO (15 μM) for 48 h, either alone or in combination. **E, F** Quiescent DU145 (**E**) and LNCaP (**F**) cells were induced to re-enter the cell cycle after 24 h of treatment with PTE (IC_{90}) and mitoTEMPO (15 μM), either alone or in combination. The percentage of apoptotic cells was assessed using Annexin V-FITC/PI-stained flow cytometry. 'Cont' refers to proliferative control cells, and 'Qsct' indicates quiescent cells. Data are presented as mean ± standard deviation. n.s., no significance, $^{**}P < 0.01$, $^{***}P < 0.001$ *versus* the indicated group.

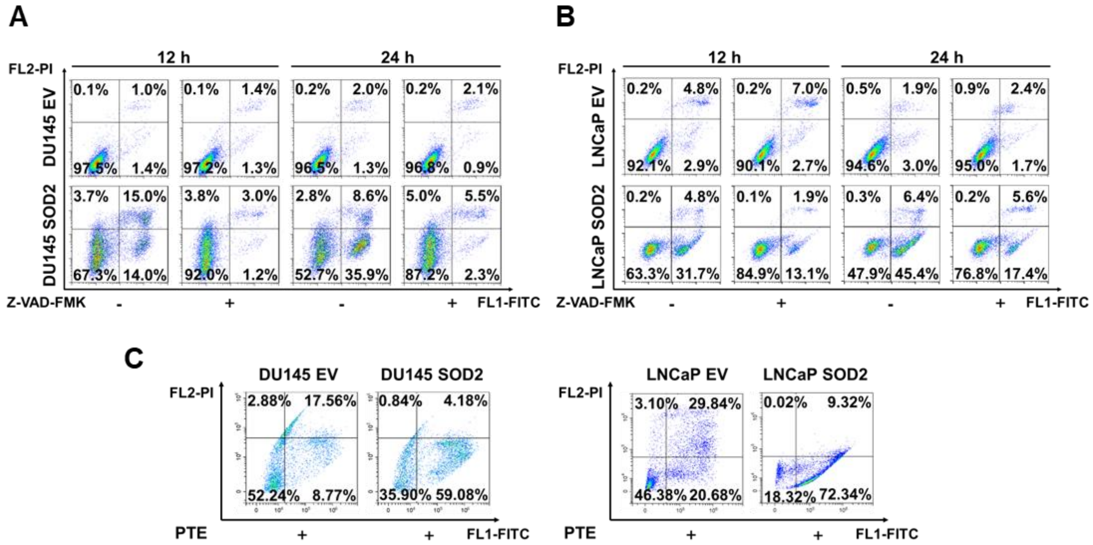

Figure S4. Quiescent EV and SOD2-overexpressing DU145 and LNCaP cells were re-activated to enter the cell cycle and treated with or without 50 μM Z-VAD-FMK (**A, B**) for 12 and 24 h or PTE (**C**) for 48 h. Representative images of apoptotic cells during cell cycle re-entry were obtained using Annexin V-FITC/PI-stained flow cytometry.

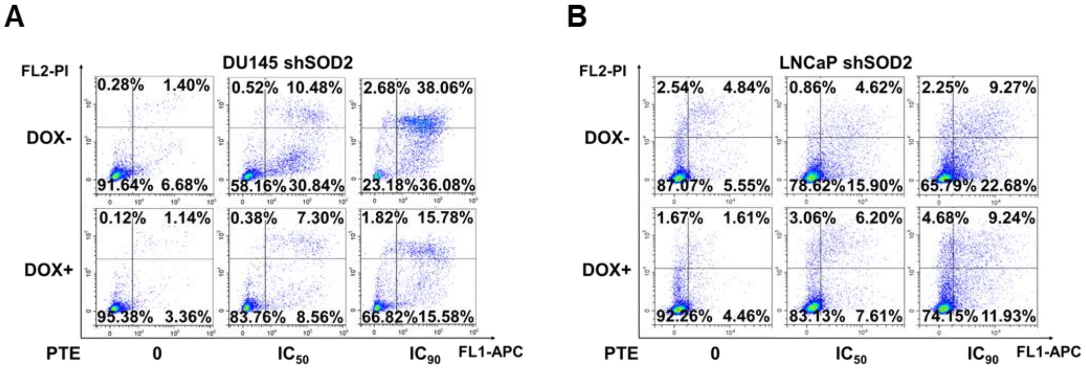

Figure S5. Re-activated quiescent DU145 shSOD2 (**A**) and LNCaP shSOD2 (**B**) cells were treated with DMSO or PTE (IC_{50} and IC_{90}) for 48 h, with or without DOX. Representative images were obtained using Annexin V-APC/PI-stained flow cytometry to assess the percentage of apoptotic cells.

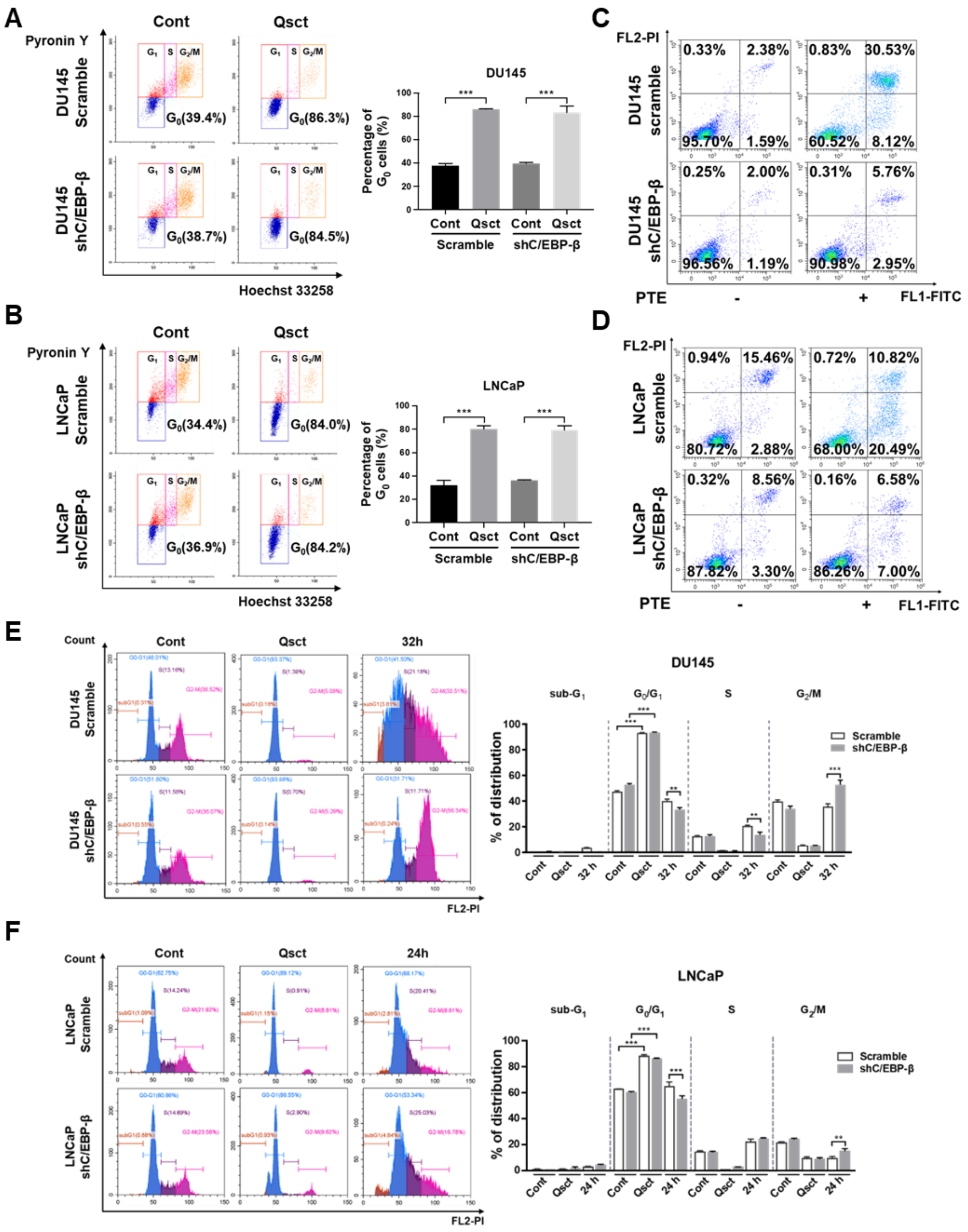

Figure S6. Knockdown C/EBP-β reduces the apoptotic effect of PTE. A, B Representative flow cytometry plots of DU145 **(A)** and LNCaP **(B)** cells with scramble control or C/EBP-β knockdown stained with Hoechst 33258 and Pyronin Y. **C, D** Representative images of Annexin V-FITC/PI-stained flow cytometry to assess apoptosis in re-activated quiescent DU145 (**C**) and LNCaP (**D**) cells with scramble

control or C/EBP-β knockdown, with or without PTE treatment for 48 h. **E, F** Quiescent DU145 **(E)** and LNCaP **(F)** cells with scramble control or C/EBP-β knockdown were induced to re-enter the cell cycle, harvested at 32 or 24 h, respectively, and subjected to flow cytometry with PI staining for cell cycle distribution analysis. Data are presented as mean ± standard deviation. $^{**}P < 0.01$, $^{***}P < 0.001$ *versus* the indicated group.
